# Comprehensive characterization of the *cis*-regulatory code responsible for the spatio-temporal expression of *olSix3.2 *in the developing medaka forebrain

**DOI:** 10.1186/gb-2007-8-7-r137

**Published:** 2007-07-06

**Authors:** Ivan Conte, Paola Bovolenta

**Affiliations:** 1 Departamento de Neurobiología Celular, Molecular y del Desarrollo, Instituto Cajal, CSIC, Dr Arce, Madrid 28002, Spain

## Abstract

**Background:**

Embryonic development is coordinated by sets of *cis*-regulatory elements that are collectively responsible for the precise spatio-temporal organization of regulatory gene networks. There is little information on how these elements, which are often associated with highly conserved noncoding sequences, are combined to generate precise gene expression patterns in vertebrates. To address this issue, we have focused on *Six3*, an important regulator of vertebrate forebrain development.

**Results:**

Using computational analysis and exploiting the diversity of teleost genomes, we identified a cluster of highly conserved noncoding sequences surrounding the *Six3 *gene. Transgenesis in medaka fish demonstrates that these sequences have enhancer, silencer, and silencer blocker activities that are differentially combined to control the entire distribution of *Six3*.

**Conclusion:**

This report provides the first example of the precise regulatory code necessary for the expression of a vertebrate gene, and offers a unique framework for defining the interplay of *trans*-acting factors that control the evolutionary conserved use of *Six3*.

## Abstract 2

A cluster of highly conserved non-coding sequences surrounding the Six3 gene were identified in fish genomes, and transgenesis in medaka fish demonstrates that these sequences have enhancer, silencer and silencer blocker activities that are differentially combined to control the distribution of Six3.

## Background

Embryonic development is coordinated by networks of evolutionary conserved regulatory genes that encode transcription factors and components of cell signaling pathways, which in many instances are repetitively exploited in space and time to generate appropriate outcomes in target cells.

Progressive specification of the vertebrate prosencephalon indeed follows this rule [1,2] and requires, among other factors, recurrent use of *Six3*, which is a member of the *Six*/*sine oculis *family of homeobox transcription factors [3]. In all vertebrates, *Six3 *is expressed from the neurula stage in the anteriormost neural plate and then in its derivatives: the developing eyes and olfactory placodes, the hypothalamic pituitary regions, and the ventral telencephalon. In mouse and chick, this distribution overlaps with that of its closely related homolog, namely *Six6 *[3]. However, with time *Six3 *and *Six6 *expressions progressively segregate to different brain regions, and *Six3 *- but not *Six6 *- is additionally expressed in the olfactory bulb, cerebral cortex, hippocampus, midbrain, and cerebellum [4]. Consistent with this expression, *Six3*-null mice die at birth, lacking most of the head structures anterior to the midbrain, including eyes [5], and mutations in *SIX3 *have been found in humans affected by holoprosencephaly and aprosencephaly/atelencephaly [6,7]. During mammalian lens induction, *Six3 *is essential in the presumptive lens ectoderm to activate *Pax6 *and possibly *Sox2 *expression [8]. In addition, morpholino-based knockdown of the medaka fish *Six3 *demonstrates the concentration-dependent need for the function of this transcription factor for proximo-distal patterning of the optic vesicles [9]. Biochemical and functional studies have also shown that *Six3*, as well as *Six6*, can induce ectopic retinal tissues and control retinal neuroblast proliferation, acting as transcriptional repressors through the interaction with members of the *groucho *family of transcriptional co-repressors [10-15]. Furthermore, Six3, but not Six6, functionally interacts with the DNA replication inhibitor Geminin, controlling the balance between cell proliferation and differentiation with a mechanism that is independent of transcriptional regulation [16].

How the activity of *Six3 *- or that of any other gene with multiple functions during embryo development - is diversified remains to be elucidated. This could be facilitated by defining the precise gene regulatory network that controls its spatio-temporal expression. It is now well established that control of gene expression is executed through sets of *cis*-regulatory regions within the noncoding DNA of animal genomes. These *cis*-regulatory modules have variable length and contain clusters of DNA-binding sites for different transcription factors. These modules work as promoter enhancers or silencers and collectively constitute a unique code for the switching on and off of gene activity [17-19].

The experimental definition of the organization of these specific *cis*-regulatory elements has progressed substantially in both *Drosophila *and sea urchin [17]. In contrast, our understanding of how these modules are combined to generate precise gene expression patterns in vertebrates is still rather limited. Possible causes of this are the increased genome complexity and the slow and laborious process of testing the functional significance of identified elements in mammals [20]. Recently, however, computational approaches based on multispecies genomic sequence alignments, combining both closely related and highly divergent organisms, have facilitated identification of highly conserved noncoding sequences, which in many cases appear to coincide with the regulatory modules of genes that play critical roles in development. Analyses of the complex regulation of genes such as *Sox2*, *Sox9*, *Otx2*, *Shh*, and *Irx *provide some illustrative examples [21-27]. Functional testing of 'enhancer' activity has also progressed, thanks to the use of alternative and relatively faster 'transgenic' approaches based on the use of nonmammalian vertebrate model systems [20,25].

Here, we have taken advantage of both the power of computational analysis and the particular compact genome and high transgenesis efficiency of the medaka fish (*Oryzia latipes*) [28] to dissect the regulatory control of one of the two *Six3 *medaka homologs, *olSix3.2*, that we identified during the course of this study. *olSix3.2 *is more closely related to the mammalian *Six3 *than the previously described medaka homolog [29] (hereafter referred to as '*olSix3.1*'). Similar to other related studies [23-25], we identified and functionally characterized sets of *cis*-regulatory modules that control the *olSix3.2 *promoter, showing that at least some of these *cis*-regulatory elements are conserved in other vertebrates, although they are dispersed over a greater stretch of DNA. Going a step further, we have also used combinations and deletions of the identified *cis*-regulatory modules to elucidate the regulatory code of *olSix3.2*, which is composed of two enhancers, two silencers, and two 'silencer blockers' used in a combinatorial manner. This comprehensive description of the *olSix3.2 cis*-regulatory code provides a unique framework for defining the network of *trans*-acting factors that control the evolutionary conserved activity of *Six3 *during forebrain development.

## Results

### Isolation, characterization, and expression of *olSix3.2*

In order to identify the elements that regulate *Six3 *expression using the medaka fish (*Oryzia latipes*) as a model, we used the available *olSix3.1 *coding sequence (AJ000937) as a query to search public databases (see Materials and methods, below) for the ortholog genomic loci of the closely related species *Fugu rubripes*, *Tetraodon nigroviridis*, and *Danio rerio *(zebrafish). This search retrieved four different loci, one for the fugu and the tetraodon, and two for the zebrafish (*six3a *and *six3b*). Alignment of about 20 kilobases (kb) of the retrieved sequences upstream of the *Six3 *translational start sites identified a cluster of conserved noncoding blocks roughly contained within the first 4.5 kb (data not shown). In the case of the zebrafish, alignment of the *six3a *or *six3b *loci yielded comparable results. This information was used to amplify from genomic DNA a fragment of the medaka *Six3 *locus that contains the corresponding conserved noncoding blocks and the entire first exon.

Interestingly, nucleotide and amino acid sequence alignment of the partially amplified *olSix3 *coding region did not completely overlap with that reported for the previously identified *olSix3.1 *[29] but identified - as in zebrafish and *Xenopus *[30,31] - a second *Six3*-related gene in the medaka genome, namely *olSix3.2 *(AM494407).

Cloning and sequencing of the entire *olSix3.2 *coding region revealed a two-exon structure, similar to that of *olSix3.1 *and the mouse *Six3*, in which the first exon encodes the *Six *and homeobox domains. *olSix3.1 *and *olSix3.2 *exhibited 76% and 63% identity at the nucleotide and amino acid levels, respectively. Interestingly, comparison of the amino acid sequence (81% versus 59%; Additional data file 1) and genomic organization, together with phylogenetic analysis (Additional data file 2), demonstrated that *olSix3.2 *was more closely related to the mammalian *Six3 *than the previously identified *olSix3.1*, which instead falls in between the *Six3 *and *Six6 *branches of the family (Additional data file 2).

**Figure 1:**
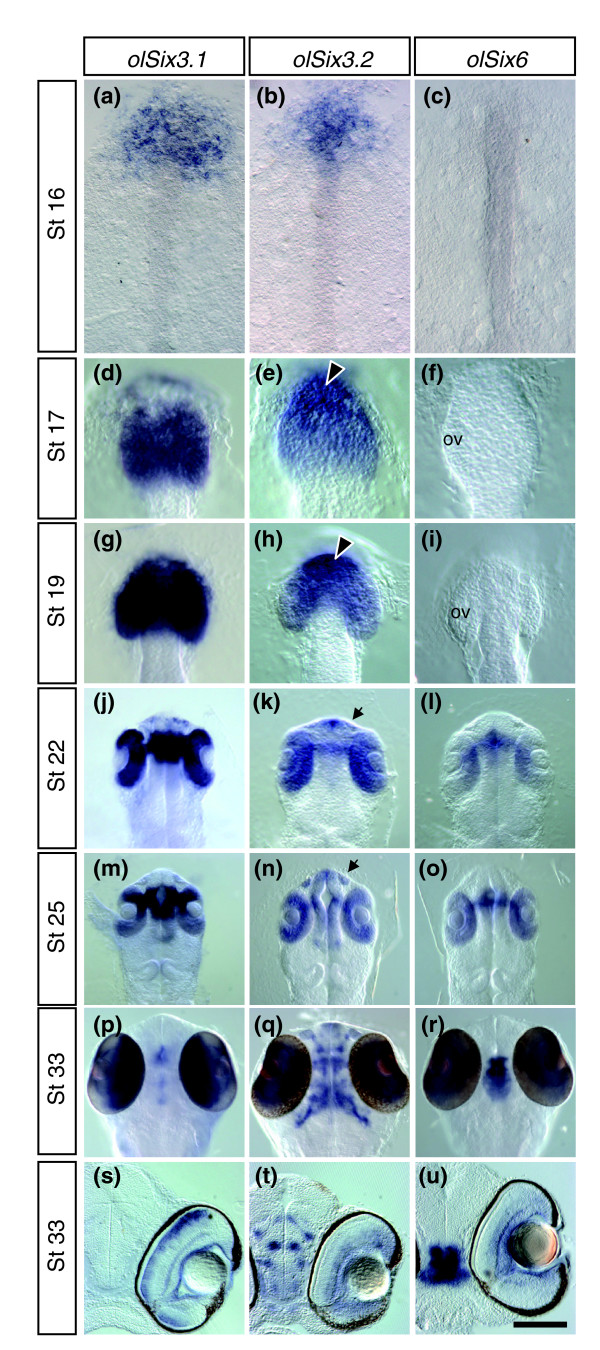
Comparative analysis of *olSix3.1*, *olSix3.2*, and *olSix6 *expression pattern during embryonic development. Medaka embryos at different developmental stages (as indicated in the panels) were hybridized *in toto *with specific probes, as indicated on the top of each column. **(a to r) **Anterior dorsal views; **(s to u) **frontal vibratome sections through the eye. From St16 to St19, only *olSix3.1 *and *olSix3.2 *are expressed in the anterior neural plate (panels a to c) and then in the presumptive telencephalon and optic vesicles (panels d to i), although *olSix3.1 *is more abundant in the optic vesicles (panels d and g) and *olSix3.2 *in the telencephalic region (arrowheads in panels e and h). From St22 onward, when *olSix6 *mRNA also becomes detectable, the three genes are co-expressed, albeit at different levels, in the developing neural retina, optic stalk, and pre-optic and hypothalamic area (panels j to r). In addition, *olSix3.2 *is distributed in the developing lens, olfactory pits (panels k and n; arrow), telencephalon, and anterior and posterior thalamus (panels k, n, and q). During retinal neurogenesis, *olSix3.2 *and *olSix6 *are restricted to the retinal ganglion and amacrine cells (panels t and u), whereas *olSix3.1 *is restricted to the inner nuclear layer (panel s).

*olSix3.1 *is expressed in the anterior embryonic shield and the developing eye [29]. To determine whether the newly identified gene and the initially identified homolog had similar distributions, we compared the expression domain of *olSix3.2 *with those of *olSix3.1 *and the related *olSix6 *[13] using whole-mount *in situ *hybridization. As for *olSix3.1*, *olSix3.2 *was first detected in the anterior neural plate at late gastrula stages but was additionally expressed in the anterior axial mesoderm at St16 (Figure 1a-c). At the optic vesicle stage, both *olSix3.2 *and *olSix3.1*, but not *olSix6*, were expressed in the forebrain. However, although *olSix3.2 *was more abundant in the presumptive telencephalon (Figure 1e,h), *olSix3.1 *was predominant in the optic area (Figure 1d,g). This distribution was more evident at later stages of development, when both *olSix3.1 *and *olSix6*, which first appears at the optic cup stage (Figure 1l) [13]), were strongly expressed in the developing neural retina, optic stalk, and preoptic and hypothalamic areas (Figure 1j,l,m,o,p,r). In contrast, *olSix3.2 *mRNA was distributed in the developing lens, olfactory pits, telencephalon, neural retina, anterior hypothalamus, and anterior and posterior thalamus (Figure 1k,n,q). During retinal neurogenesis, *olSix3.1 *was mostly confined to the inner nuclear layer (Figure 1s), and *olSix3.2 *and *olSix6 *to the retinal ganglion and amacrine cells (Figure 1t,u).

In conclusion, the distribution of *olSix3.2 *appeared closely related to that reported for the chick and mouse *Six3 *[4,32,33], whereas the combined expression patterns of *olSix3.1 *and *olSix6 *resembled that reported for *Six6 *[34,35].

### The *cis*-regulatory elements responsible for *olSix3.2 *expression are contained in a 4.5 kb genomic region ending with a distal 'silencer'

**Figure 2:**
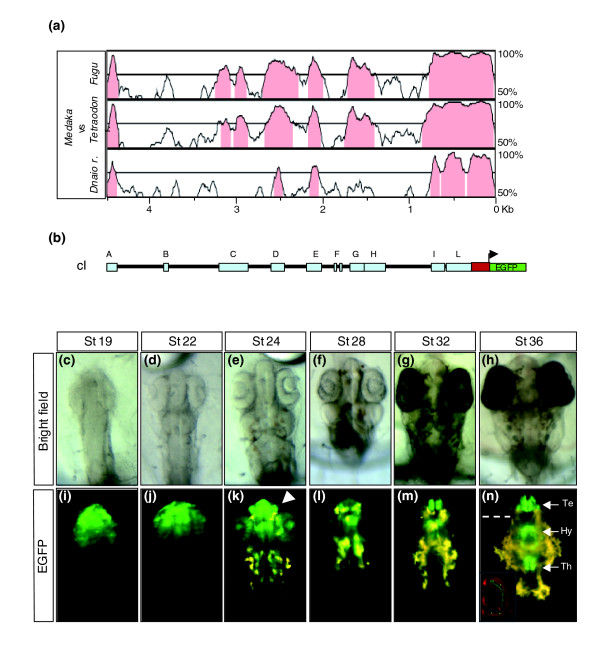
The *cis*-regulatory elements responsible for the *olSix3.2 *expression are contained in a 4.5 kb genomic region. **(a) **VISTA comparison of the 5' *olSix3 *genomic region plotted against those from *Fugu rubripes*, *Tetraodon nigroviridis*, and *Danio rerio*. The blocks of sequences (75% identity over 100 base pairs) conserved among the four species are indicated in pink. **(b) **Schematic structure of the 5' *olSix3.2 *genomic region/enhanced green fluorescent protein (EGFP) reporter construct (cI) containing ten highly conserved noncoding regions represented as light blue rectangles A to L. The red rectangle represents the 5'-untranslated region and the first nine nucleotides of the *olSix3.2 *coding sequence in frame with a nuclear *EGFP *reporter (green). **(c to h) **Bright field images; and **(i to n) **epi-fluorescence dorsal views of cI transgenic embryos at different stages of development (as indicated). Note that the cI construct drives EGFP reporter expression to the same *olSix3.2 *expression domain, recapitulating its entire pattern (compare with Figure 1). The arrowhead in panel k points to the olfactory pits. The inset in panel n shows a frontal section through the eye (dotted line), where EGFP is expressed in the amacrine cells. The section was counter-stained with propidium iodine (red). Hy, hypothalamus; Te, telencephalon; Th, thalamus.

On the basis of this expression pattern, we next searched for the elements that could be involved in the regulation of *olSix3.2 *expression. Alignment of the amplified *olSix3.2 *genomic sequence with the corresponding sequences from fugu, tetraodon, and zebrafish (analyses involving *six3a *and *six3b *yielded similar results) identified ten conserved noncoding blocks within the 4.5 kb upstream of the translational start site *olSix3.2 *(Figure 2a).

Owing to selective pressure, functional elements in genomes evolve at a slower pace than nonfunctional regions [36-39]. A number of recent studies have functionally demonstrated that a proportion of the highly conserved noncoding regions present in vertebrate genomes correspond to regulatory elements with enhancer activity [21,39]. We therefore asked whether the region containing the cluster of ten highly conserved noncoding elements was necessary and sufficient to control the entire expression of *olSix3.2*.

To this end we fused this 4.5 kb genomic region, including the first nine nucleotides of the coding sequence, in frame with a nuclear *EGFP *(enhanced green fluorescent protein) reporter (Figure 2b). This construct, containing the ten conserved noncoding blocks (termed A-L; Figure 2b), was used to generate three independent stable transgenic medaka lines, which all exhibited a spatio-temporal distribution of the reporter virtually identical to that observed for the endogenous *olSix3.2 *both at embryonic (compare Figure 1 with Figure 2c-n) and adult stages (not shown). We thus concluded that this region was sufficient to control the entire expression of *olSix3.2*.

In addition to regulatory elements, sequence conservation could reflect the existence of natural anti-sense mRNAs [40] or of alternative and yet uncharacterized exons of *Six3*. However, reverse transcription polymerase chain reaction (RT-PCR) analysis and *in situ *hybridization studies excluded these possibilities (data not shown). We thus assumed that the ten modules, identified on the basis of their conservation among teleosts (the precise nucleotide sequence of each module is provided in Additional data file 3), could all potentially contain elements that are involved in the regulation of *olSix3.2*. To test whether this assumption was correct, we generated a series of constructs (named cI to cXXVII) carrying different combinations of the A-L modules, which were then functionally assayed by generating and analyzing three independent stable transgenic lines for the vast majority of the constructs. In each case, the pattern of expression of the *EGFP *reporter was compared with that observed with construct I (cI), containing the full 4.5 kb sequence (Figure 2i-n) and was always consistent with that observed in F_0 _injected embryos.

**Figure 3:**
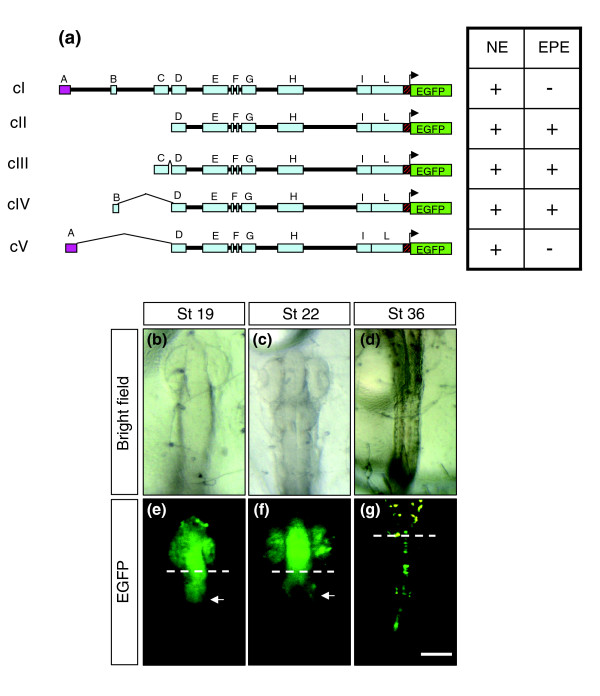
The most distal conserved module, A, is a silencer that restrains *olSix3.2 *expression to the anterior neural plate. **(a) **Drawings to the left of the panel are schematic representations of the different constructs (cI to cV) used to study the potential regulatory activity of modules A to C, whereas the tables to the right summarizes the presence (+) or absence (-) of enhanced green fluorescent protein (EGFP) reporter expression observed with each construct and corresponding to the endogenous *olSix3.2 *expression domain (NE) or with an ectopic posterior expansion (EPE). The A module with silencer activity is depicted in purple. **(b to d) **Bright field images, and **(e to g) **epi-fluorescence dorsal views of cII transgenic embryos at different stages of development (as indicated). Note that the domain of EGFP expression is progressively expanded in the caudal direction (arrows in panels e and f), invading the spinal cord at St36 (panel g). Equivalent patterns were observed with the cIII and cIV transgenic lines. Dotted lines in panels e to g indicate the caudal limit of endogenous *olSix3.2 *expression.

Embryos of a transgenic line carrying a construct in which the A to C modules had been deleted (cII; Figure 3a) showed a pattern of *EGFP *expression in the anteriormost neural tube similar to that observed with cI. However, embryos consistently exhibited an additional transient expansion of *EGFP *distribution to posterior mesencephalic regions (compare Figure 3e,f with Figure 2i,j and Figure 1h,k), which disappeared after St22. EGFP fluorescence was also consistently observed in the spinal cord starting from St34 (Figure 3d,g) up to adult stages. These observations suggested that, presumably, blocks D to L were sufficient to control normal *olSix3.2 *expression, whereas the A to C modules contained a silencer(s), the activity of which was necessary to restrain *olSix3.2 *expression to anterior domains of the neural tube throughout development. To determine the location of the silencer activity, we generated and functionally analyzed three different constructs containing the D to L modules in combination with the A, B, or C block (cIII to cV; Figure 3a). Only the presence of 134 base pairs (bp) of the A module could repress the posterior *EGFP *expansion, restoring the normal *olSix3.2 *distribution, which clearly identified the presence of a *cis*-regulatory silencer(s) in this sequence. In spite of sequence conservation, the B and C blocks instead did not appear to contribute to the spatio-temporal control of *olSix3.2*, at least in the context that we tested.

### Early expression of *olSix3.2 *in the anterior neural structures depends on one enhancer, whereas that in the lens placode requires the additional activity of four *cis*-regulatory modules

**Figure 4:**
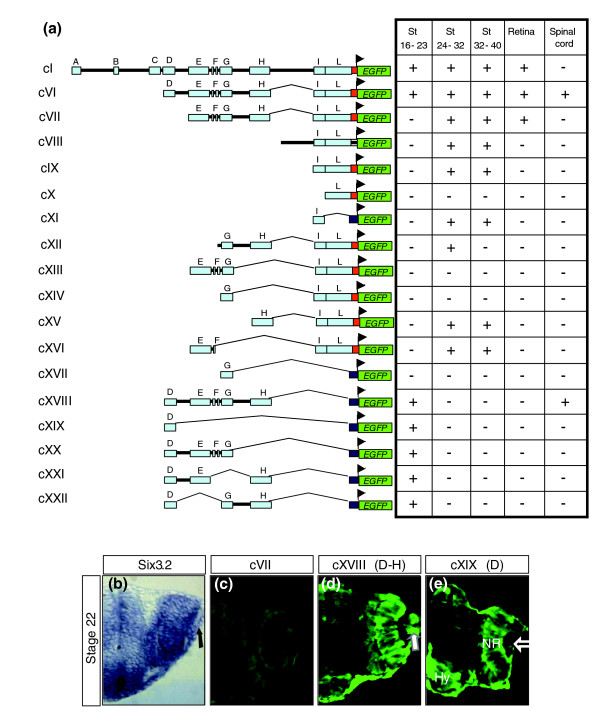
Different constructs used to generate stable transgenic lines and corresponding distribution of EGFP reporter in expected *olSix3.2 *expression domains. **(a) **Drawings to the left of the panel are schematic representations of the different constructs (cI and cVI to cXXII) used to generate stable transgenic lines, whereas the tables to the right summarize the presence (+) or absence (-) of enhanced green fluorescent protein (EGFP) reporter expression corresponding to the expected *olSix3.2 *expression domain at different stages of differentiation, in the retina or ectopically in the spinal cord. The red box represents the 5'-untranslated region and the first nine nucleotides of the *olSix3.2 *coding sequence, in frame with a nuclear *EGFP *reporter, whereas the dark blue box represents the minimal tyrosine kinase promoter. **(b to e) **The images show frontal vibratome sections through the optic cup of *in situ *hybridized **(b) **wild type and **(c) **cVII, **(d) **cXVIII and **(e) **cXIX transgenic lines. Note that module D alone is sufficient to drive EGFP expression in the hypothalamus and neural retina but not in the lens (empty arrow in panel e), whereas in its absence *EGFP *expression is completely lost (panel b). A similar absence of *EGFP *expression was observed in the cVIII to cXVII transgenic lines, all of which lack module D. Note also that the combination of modules D to H is necessary for expression in the lens placode (arrow in panel d), as indicated by *in situ *hybridization of the endogenous *olSix3.2 *distribution (arrow in panel b). Hy, hypothalamus; NR, neural retina.

We then sought to determine the functional relevance of the remaining D to L conserved modules. To this end we generated a series of additional constructs (named cVI to cXXII; Figure 4a) based on selective deletion of one or more modules at the time or by including different combinations of a few of them. Transgenesis analysis of these constructs demonstrated that the D module was necessary (cVI to cXVII; Figure 4a,c) and sufficient (cXIX; Figure 4a,e) to drive EGFP expression in all of the anterior neural structures from St16 to St23. In contrast, the D module was necessary but not sufficient (cXIX; Figure 4e) to control *EGFP *expression in the lens placode/lens vesicle, as normally observed for the endogenous *olSix3.2 *(Figure 4b). Indeed, the activity of modules E to H was further required for EGFP expression in the lens (cVI and cXVIII; compare Figure 4d with Figure 4e), because deletion of either one of them was sufficient to abrogate the reporter expression in the lens ectoderm (cXIX to cXXII; Figure 4a,e), suggesting that multiple *cis*-regulatory sequences spread along these four modules contribute to *olSix3.2 *expression in this tissue. This is somewhat in contrast with the apparently simpler regulation of *olSix3.2 *distribution in the early neural tissue, which mostly depends on the D block.

Notably, modules D to H (cXVIII; Figure 4a) were also sufficient to induce caudal expansion of reporter expression, with a pattern identical to that observed in the absence of the A module (Figure 3e-g), indicating that modules I and L do not contribute to this expansion or to early expression of the gene.

### During organogenesis, appropriate expression of *olSix3.2 *requires the combined activity of two silencers, one enhancer, and two putative 'silencer blockers'

**Figure 5:**
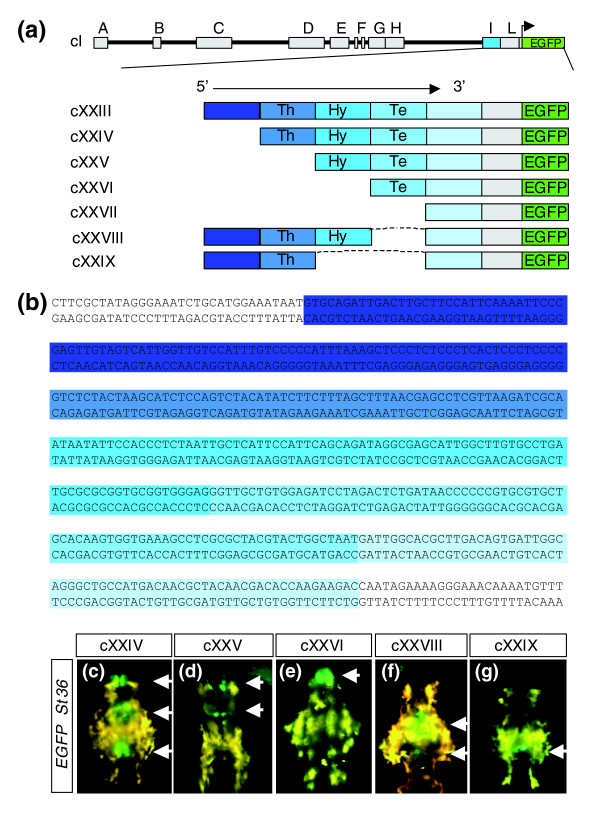
Module I contains a 5' to 3' organized sequence of *cis*-regulatory elements that control the posterior to anterior expression of *olSix3.2 *in brain. **(a) **The drawings illustrate the design of the cXXIII to cXXIX constructs use to determine the arrangement of the *cis*-regulatory elements within module I, using five progressive deletions of about 50 base pairs, indicated by a gradient of blue colors. **(b) **Nucleotide sequence of module I, in which the precise position of the deletions is indicated with the same gradient of blue colors. **(c to g) **Epi-fluorescence dorsal views of cXXIV to cXXIX transgenic embryos that show the loss of thalamic (panel d), hypothalamic (panels e and g), and telencephalic (panels f and g) reporter expression. cXXVII transgenic embryos exhibited no enhanced green fluorescent protein (EGFP) expression. Hy, hypothalamus; Te, telencephalon; Th, thalamus.

To determine whether these last two modules were functionally relevant to any other aspect of *olSix3.2 *expression, we designed a number of constructs in which modules I and L were assayed separately (cX and cXI), in conjunction (cIX), and combined with the *olSix3.2 *endogenous promoter (cX) or with the minimal tyrosine kinase promoter (cXI). Injections of cX were not associated with *EGFP *expression in any region of the embryo at any stage (Figure 4a). This indicates that, as in the case of modules B and C, the L block had no enhancer silencer activity relevant to the regulation of *olSix3.2*, at least in the tested conditions, although its sequence is strongly conserved among all vertebrates. In contrast, the activity of block I was clearly linked to control of *olSix3.2 *distribution in the forebrain starting from St26 onward, when *EGFP *was gradually observed, with progressively increasing intensity, first in the telencephalic, then in the hypothalamic, and finally in the thalamic region (Figures 4a and 5c). This recapitulates the endogenous expression of the gene (Figure 1q).

To determine the minimal region of module I involved in the control of this expression, we engineered five 5' to 3' stepwise deletions covering the entire module (cXXIII to cXXVII; Figure 5a). Notably, deletions two, three, and four resulted in progressive abrogation of *EGFP *expression in the thalamic, hypothalamic (Figyre 5b-d), and telencephalic regions (not shown). This strongly suggests that module I contains a 5' to 3' organized succession of *cis*-regulatory elements that control the posterior to anterior spatio-temporal organization of *olSix3.2 *expression in the developing brain. This interpretation was further supported by the injection of two internal deletion constructs (cXXVIII and cXXIX) in which the stretches of nucleotides apparently responsible for hypothalamic and telencephalic expression were removed from cXXIII (Figure 5a). Indeed, in 11% (close to transgenic efficiency) of the embryos analyzed in F_0_, EGFP fluorescence was not detected in the telencephalon (cXXVIII; Figure 5f) or in the hypothalamus and telencephalon (cXXIX; Figure 5g), clearly indicating that deleted elements are the main driver of *olSix3.2 *expression in these regions.

**Figure 6:**
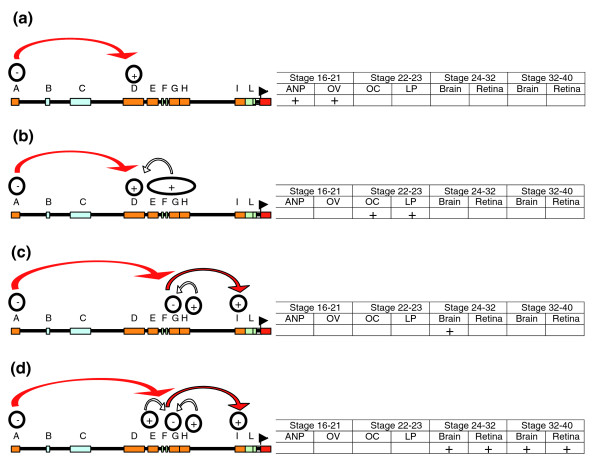
Summary of the regulatory code that control the entire expression of *olSix3.2*. **(a) **Early expression of *olSix3.2 *in the forebrain and eye depends on enhancers in module D and a silencer activity (activities) in module A. **(b) ***olSix3.2 *expression in the lens placode requires multiple elements distributed along modules D to H. **(c) **During organogenesis, correct *olSix3.2 *expression requires the activity of different enhancer arranged in a 5'to 3' mode within module I. The activity of I is repressed by module G, which, in turn, is neutralized initially by module H and at later stages **(d) **by the combined activity of the E and H silencers. Module A is necessary at all stages analyzed to prevent reporter expansion to caudal central nervous system.

The elements contained in the I module appeared to suffice in terms of regulating late *olSix3.2 *embryonic expression in the brain. Nevertheless, we considered whether any additional module could modify their activity. Transgenic embryos carrying cXIV, in which the G module was combined with the I module, had no reporter expression in the brain (Figure 4a), raising the possibility that the G module contained a 'silencer' that, in turn, could be normally regulated by a 'silencer blocker', as previously proposed [41,42]. Addition of the H block (cXII) proved that this was the case, because its presence restored reporter expression, although only from St26 to St32. Further addition of the E block (cVII, containing E, G, H and I) appeared to overcome the effect of the G silencer from St32 onward. Thus, proper regulation of late *olSix3.2 *embryonic expression requires the participation of five different modules - one enhancer, one silencer, and two silencer blockers - in addition to the silencer activity contained in the distal A module (Figure 6c,d).

When tested alone, block I did not drive *EGFP *expression in the differentiating retina, whereas activity of the D block was sufficient to maintain reporter expression only in the prospective neural retina (Figure 4a,d,e). Thus, *olSix3.2 *expression in the differentiating retina appeared to depend on a combination of modules different from those tested thus far. The search for this code demonstrated that only the combined activity of the E to I modules (cVII; Figure 4a) was effective in supporting EGFP expression in the late developing retina.

### Identification and characterization of conserved regions among vertebrate

Altogether these data provide a detailed picture of the regulatory code that governs *olSix3.2 *expression during eye and brain development in medaka. As summarized in Figure 6, this spatio-temporal code is provided by the combined use of at least seven different modules, all conserved among fishes, with distinct enhancer, silencer, or silencer blocker activities. The next logical question was whether this regulatory organisation was conserved in the *Six3 *locus of vertebrates other than fishes.

**Figure 7:**
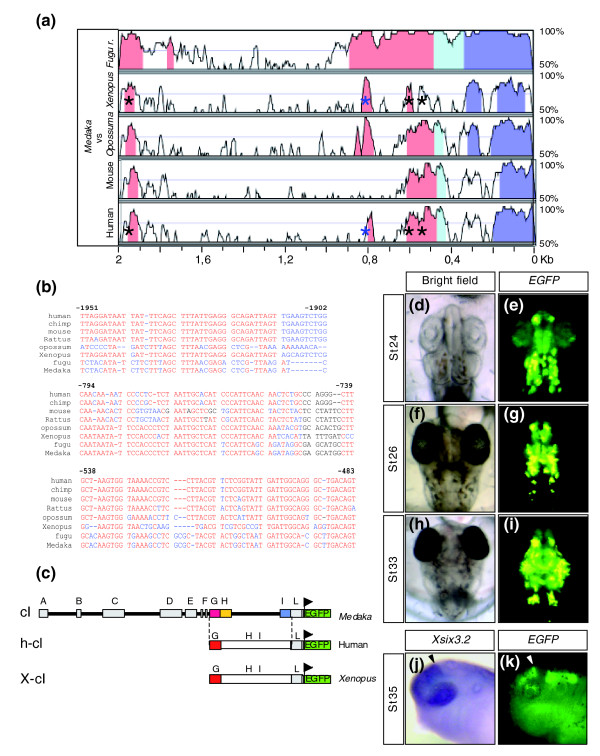
Modules G, H, and I are functionally conserved in humans. **(a) **VISTA comparison (90% identity over 25 base pairs) of the medaka *olSix3.2 *genomic region plotted against those of other vertebrates, as indicated. The analysis identifies highly conserved noncoding regions (pink peaks) corresponding to modules G (asterisk) and L (two asterisks), and to a partial I element (blue asterisk). The light and dark blue peaks correspond to the 5'-untranslated region and coding sequence of *Six3*, respectively. **(b) **Nucleotide sequence alignment of module I from different vertebrates where partially or completely conserved sequences are indicated in blue or red, respectively. Nonconserved sequences are in black. The nucleotide positions are relative to the human genomic sequence. **(c) **Schematic representation of the human (h-cI) and *Xenopus *(X-cI) constructs, containing the G (red box), H, and I sequences, used to generate transient *Xenopus *and stable transgenic medaka lines. The mixed H and I sequences are represented as a striped blue and yellow box. **(d, f, and h) **Bright field images and **(e, g, and i) **epi-fluorescence dorsal views of h-cI transgenic medaka embryos at different stages of development, as indicated in the panels. **(j and k) **Lateral views of St35 *Xenopus *embryos hybridized with a specific probe for *Xsix3.2 *or injected with X-cI. Note that in both *Xenopus *and medaka embryos reporter expression recapitulates *Six3 *expression at the corresponding stages of development.

To address this problem, we used the characterized *olSix3.2 *regulatory region as a query to search public databases (Genome Bioinformatics UCSC [University of California, Santa Cruz]) for the ortholog regions in vertebrates other than fishes. This analysis showed that only part of the modules identified in teleosts were conserved among all vertebrate phyla (Figure 7a). Attempts to align each of the A to F modules separately and enlarging the search to the 120 kb flanking *Six3 *in the *Xenopus laevi*, chicken, mouse, and human genomes were unsuccessful in detecting alignable sequences using the VISTA and multialign software [43,44]. Thus, only the G and L modules were highly conserved and similarly organized in all genomes, whereas the sequences that constitute the H and I modules in fishes were conserved but fragmented in a larger stretch of DNA in the other genomes analysed (Figure 7b), with the exception of the marsupial opossum, in which the I block was co-linear with that of fishes (data not shown). In spite of fragmentation, transgenic embryos, carrying the human sequence that included the G module and the dispersed H and I sequences (Figure 7c), exhibited spatio-temporal *EGFP *expression in the developing brain identical to that observed in the equivalent medaka genomic region (Figure 7d-i). In addition, reporter expression was observed in the lens placode/vesicle. This suggested that although control of at least part of *Six3 *expression in the brain has been conserved, its regulation during lens development has undergone a reorganization of the appropriate *cis*-regulatory elements during evolution (data not shown).

Although the human construct (h-cI) we injected drove *EGFP *expression only in the late *olSix3.2 *expression domain, according to what expected given the sole presence of modules G to L, we could not exclude that this human region contained regulatory information not readable in fish. Thus, to rule out possible cross-species interferences, we amplified from genomic DNA the equivalent *Xenopus *region, in which the G to L elements are organized as in humans (Figure 7a). Transgenesis analysis in *Xenopus *embryos using a construct containing this fragment (X-cI; Figure 7c) yielded results equivalent to those observed with the human fragment; EGFP reporter expression was detected only at later stages of brain development in the expected domain of *Xsix3.2 *expression (Figure 7j,k). This supports the idea that the regulatory information for early *Six3 *expression in vertebrates other than fishes reside in as yet unidentified genomic regions.

## Discussion

*Six3 *is an important regulator of vertebrate forebrain development. Gene regulatory network models predict that the precise spatio-temporal expression pattern of genes fundamental for embryo development must be orchestrated by the interaction of various regulatory regions [17]. Supporting the model, we functionally demonstrated that the entire expression of the newly identified *olSix3.2 *is orchestrated by the combined use of seven different *cis*-regulatory modules (Figure 6) and that at least part of this regulation is conserved in the *Six3 *locus of vertebrates other than fishes. Two main 'enhancer' modules (D and I) are responsible for *olSix3.2 *expression at early and late stages of brain development, respectively. Their activity is spatially refined by the function of two 'silencers' and two 'silencer blockers'. In addition, *olSix3.2 *expression in the lens ectoderm and in the differentiating retina requires the combined activity of five different *cis*-regulatory modules. This apparently simple regulation may hide additional organization, as we have demonstrated for the I enhancer, in which an organized sequence of *cis*-regulatory elements control the posterior to anterior expression of *olSix3.2 *in the brain.

The availability of different genome sequences and the development of analytical bioinformatic tools have facilitated study of *cis *regulation of a number of genes with evolutionary conserved roles in vertebrate embryonic development. Some of these studies have focused, as has ours, on a specific gene or a gene cluster, identifying enhancers that are involved in the control of specific expression domains [21,23,25,45-49]. However, possibly because of the size of the genomic regions that are involved, or to the laborious and time consuming use of mice, or the limitations of chick electroporation in validating regulatory activities, these studies have mostly focused on each enhancer as a separate entity, thus missing the effects of possible cooperative activities. Other recent and extremely informative studies, based on medium or small throughput screens in zebrafish, have instead systematically tested the autonomously enhancing function of large numbers of highly conserved noncoding elements positioned in areas surrounding developmentally important genes, with positive identification only of a fraction of them [21,39]. Because each element is tested in an unconstrained context, negative regulators as well as modulatory functions of surrounding endogenous elements are also undetected using these approaches [39]. In contrast, possibly benefiting from the high transgenesis efficiency of the medaka fish [50] and its compact genome, we were able to assign enhancer, silencer, and modulatory functions to the majority of the highly conserved noncoding elements surrounding the *Six3 *gene in fishes. Testing different combinations of these elements, we have also established their required interactions for proper expression of the gene. Thus, to our knowledge, we provide the first description of the regulatory code necessary for the expression of a vertebrate gene and offer a unique framework to define the entire interplay of *trans*-acting factors that control the evolutionary conserved use of *Six3 *during forebrain development.

Teleosts are the most diverse class of vertebrates with a huge variety of different species; they are characterized by broad size range and dynamic organization of genomes, which are the result of an initial genome duplication followed by subsequent independent evolution of the different lineages [51,52]. Comparison of divergent teleost genomes largely separated in the phylogenetic tree, such as the medaka and zebrafish genomes (approximately 115 to 200 million years [28]), is thus a powerful tool with which to study gene regulatory mechanisms. Adopting this strategy, we identified a cluster of potential regulatory modules in the *Six3 *locus, which were barely identifiable in a comparison among mammalian genomes (compare Figure 2a with Figure 7a). VISTA analysis of the available genomic sequences flanking the homologous vertebrate *Six3 *genes revealed several blocks of highly conserved noncoding sequences in the gene surroundings. Although a few of these blocks were located downstream of the coding sequence (data not shown), we demonstrated that the pattern of *olSix3.2 *expression could be recapitulated by 4.5 kb of genomic sequence flanking the 5' end of the gene. This conclusion is based on a relatively efficient (roughly 20% of injected embryos) and highly reproducible (basically 100%, albeit with different EGFP intensity, thus excluding chromosomal position effects) transgenic analysis using three independent and stable medaka lines generated for all of the constructs we tested. Thus, we are fairly confident that we identified the main regulatory region for *olSix3.2*, although we cannot entirely exclude the possibility that additional or duplicated regulatory elements positioned in untested regions may contribute to a refinement of the main expression domain. Indeed, redundant *cis*-regulatory elements have been reported to control specific expression domains in different genes, including *Otx2*, *Shh*, and *Sox2 *[22,23,25].

According to our analysis, the regulatory region of *olSix3.2 *is relatively compact as compared with those reported for other genes that are involved in neural development, such as *Sox2*, *Sox9*, *Otx2*, *Pax6*, and *Shh*, for which enhancers located in the region of 10, 100, and even 1,000 kb away from their promoters have been reported [23-27,53,54], even in the compact *Fugu *genome [46]. Genes with complex patterns of expression are predicted to have more regulatory elements and occupy significantly more space in the genome than those with simpler expressions that are restricted to populations of cells with similarities or shared identity [55]. It is thus possible that the compactness of the *olSix3.2 *regulatory region might reflect the association that exists among the main territories in which the gene is expressed. Indeed, the specification of telencephalic and eye fields appears to be closely linked [2], and the initial expression of *olSix3.2 *in both regions appears to depend on the activity of a single enhancer element (D) and a distal silencer (A), which constrains the expression domain to the anteriormost neural tube. This hypothesis could also explain why the combined activities of five different modules (D to H) are instead needed to control expression in the lens placode, which is the only non-neural domain of *olSix3.2 *expression. Nevertheless, compactness does not appear to be, at least in this case, a reflection of simplicity, because each of the conserved modules may include additional regulatory organization. This is the case of module I, which is the main enhancer involved in the late embryonic expression of the gene. Stepwise and internal deletions of this module have revealed a peculiar organization, in a 5' to 3' direction, of a series of *cis*-regulatory elements that are required for the posterior to anterior spatio-temporal expression of *olSix3.2 *in the thalamus, hypothalamus, and telencephalon. The activity of the I module is refined by a silencer, G, the activity of which is modulated by two silencer blockers that act in a temporal sequence, thus establishing an elaborate control code. Furthermore, although the L module *per se *has no activity, we cannot totally exclude the possibility that this module might contribute, together with modules E to H, to the regulation of I, because it was present in the constructs used for this analysis.

Alternatively, the short-range regulation of *olSix3.2 *may be linked to the chromosomal localization of the *Six *genes, which are organized in two evolutionarily conserved clusters [56]. Although the expression of the other *Six *family members (*Six1*, *Six2*, *Six4*, and *Six5*) is mostly associated with tissues of mesodermal and ectodermal origin [3], it is possible that genes within the same cluster (*Six4*, *Six1*, and *Six6*) will share a few regulatory elements, which might have imposed constrains against rearrangement during evolution [57].

*In silico *comparison identified ten conserved modules in the teleost *Six3 *locus. Transgenic analysis in medaka demonstrated clear regulatory activity for seven of them, whereas modules B, C, and L did not influence *EGFP *reporter expression. Although these modules might have subtle regulatory activities below the resolution of our analysis, their conservation could reflect other important roles in gene transcription control, such as regulation of chromatin structure or - in the particular case of module L - they may contribute to minimal promoter functions.

The regulatory region we have studied belongs to a newly identified medaka *Six3 *gene, namely *olSix3.2*. Genomic organization and phylogenetic analysis suggests that *olSix3.2 *is more closely related to the mammalian *Six3 *than the previously identified *olSix3.1 *[12]. Like its mammalian homolog [4], *olSix3*.2 is strongly expressed in various forebrain regions where its paralog is not expressed. Our comparative expression study suggests that the combination of expression domains of *olSix3.1*, *olSix3.2*, and the related *olSix6 *correspond to the combined tissue distribution observed for the mouse and chick *Six3 *and *Six6 *[32-34], with a preponderant expression of *olSix3.1 *in the eye, of *olSix3.2 *in the telencephalic and thalamic regions, and of *olSix6 *in the hypothalamus. Genetic abrogation studies in mice demonstrated that *Six3 *is necessary for the formation of forebrain, which is absent in homozygous embryos [5]. Genetic deletion of *Six6 *instead is associated with pituitary defects, absence or hypoplasia of the optic nerves, and chiasm and alteration in neural retina proliferation [58]. How the functions of *olSix3.1*, *olSix3.2*, and *olSix6 *relate to those described in the mouse for *Six3 *and *Six6 *is still unresolved and knock-down analysis of all three genes in medaka will be necessary to address this issue. Thus far, morpholino-based knock-down of *olSix3.1 *results in forebrain and eye defects, including loss of optic stalk markers [9], whereas preliminary analysis indicates that *olSix3.2 *morphants are characterized by strong ventral forebrain defects with minor eye malformations (De la Torre A, Conte I, Bovolenta P, unpublished observations), suggesting that the two *olSix3 *paralogs may cover *Six3 *as well as part of the mouse *Six6 *functions, a possibility that is also supported by the phylogenetic position of olSix3.1, which falls almost in between the *Six3 *and *Six6 *branches of the *Six *gene family (Additional data file 2).

Comparative analysis of the regulatory code of the three medaka genes currently ongoing in our laboratory might be useful in complementing these studies by providing insights into the sub-functionalization or neo-functionalization of *olSix3.1*, *olSix3.2*, and *olSix6 *as compared with their mammalian counterparts. Furthermore, they will help to elucidate whether *Six3 *and *Six6 *have arisen from the duplication of a common ancestor, as previously proposed [56], possibly duplicating at least part of their regulatory region. This is an important point because, with the comparison parameters used, we were unable to identify in other vertebrate species the conservation and distribution of the A to F regulatory modules characterized in fishes. This is particularly important for the A and D modules, which are the main regulators of early *Six3 *expression in fishes. Informatics searches of corresponding regions in mammalian genomes yielded no clear information, suggesting that these modules might be present outside the regions that we analyzed or they might have evolved differently in other vertebrate genomes, making their search even more difficult than that of the H and I modules. Alternatively, these modules may represent a new acquisition of *olSix3.2 *caused by teleost genome duplication.

In our study, we demonstrated strong functional conservation between fishes and other vertebrates only for the G, H, and I modules, which control late expression of *olSix3.2*. The sequences that compose the H and I modules in fishes were intermixed and differently arranged in other vertebrate genomes, although their function was strongly conserved when assayed in medaka and *Xenopus *transgenesis. This suggests that sequences from different vertebrates are activated by common transcription factors, although the binding sites for these factors might be distributed, oriented, or represented in different numbers among species. An additional explanation for the different arrangement of the H and I modules might be species-specific nucleotide modifications, which have been proposed to contribute to gene transcriptional evolution [59-61].

Conservation of regulatory function between human and fish in the absence of clear sequence conservation has previously been reported also for the *RET *gene. In this case, lack of correlation between the two events was even more marked, and different *in silico *analysis designed to detect shorter stretches of sequence similarities or the existence of inversion and rearrangement failed to detect alignable sequences [62]. Thus, our data, together with few additional observations [63-65], strongly support the idea proposed by Fisher and colleagues [62] that some relevant regulatory information might be conserved among species at a level that is not detectable using genomic sequence alignment.

## Conclusion

Our study established the *cis*-regulatory code required for the proper expression of *olSix3.2 *and demonstrates that there is a need to test different combinations of highly conserved putative *cis*-regulatory regions to elucidate how each conserved element contributes to the spatio-temporal control of gene expression. In fact, one limitation of previous studies that have used transgenic analysis to test the function of highly conserved noncoding sequences is the identification of single enhancers uprooted from possible interactions with the remaining regulatory elements. Our comprehensive description of the *olSix3.2 *regulatory code is now a powerful starting point from which to define the entire interplay of *trans*-acting factors that control the evolutionarily conserved use of *Six3 *during forebrain development. From a broader perspective, this type of information will be necessary to elucidate the composition and evolution of vertebrate gene regulatory networks, as compared with those of invertebrates such as *Drosophila *and sea urchin, in which this type of information is accumulating at a much faster pace [17].

## Materials and methods

### Microinjection and establishment of transgenic lines

Adult and embryonic medaka fishes (*Oryzia latipes*) from the Cab inbred strain were used throughout the study. Fertilized eggs were collected immediately and incubated at 4 to 10°C in Yamamoto's embryo rearing medium to suppress further development [66]. DNA was prepared using a High Pure Plasmid Isolation Kit (Roche, Basel, Switzerland). DNA injections (10 ng/μl DNA in ISceI enzyme reaction) were performed as previously described [50]. Embryos were staged according to the method proposed by Iwamatsu [66], raised to sexual maturity, and transgenic founder fishes were identified by out-crossing to wild-type fishes. Transcriptional activation of the constructs was monitored by EGFP expression observed in living embryos under UV fluorescent stereo-microscopy (Leica Microsystems, Wetzlar, Germany). *Xenopus laevis *embryos were obtained and raised as described previously [21]. *Xenopus *transgenesis was performed following the same procedures as used for the medaka embryos.

### Whole-mount *in situ *hybridization

Whole-mount in situ hybridizations were performed as previously described using digoxigenin labelled riboprobes [29]. Anti-sense and sense riboprobes for medaka *olSix3.1*, *olSix3.2*, and *olSix6 *and the *Xenopus Xsix3.2 *were used. A minimum of 40 embryos were hybridized for each marker and condition. *In toto *hybridized embryos were photographed, embedded in gelatine/albumine block, and further sectioned using a vibratome (Leica Microsystems, Wetzlar, Germany).

### Sequence analysis

The vertebrate *Six3 *genomic sequences were retrieved from public databases: Genome Browser UCSC [67] and JGI [68]. The genomic sequence of *olSix3.2 *was isolated from medaka genomic DNA using the following primers: *olSix3 *forward CCTCATTAAATGTCGCTAAC, and *olSix3 *reverse cgcctaatgacac cagcctc. Sequence alignments were performed using the VISTA [43] and Multalign programs [44], which are available at the corresponding websites [69,70]. The criterion used for comparisons was a minimum 75% nucleotide identity with a window size of over 100 bp. Phylogenetic analysis was performed using the PHYLIP package [71]. The results were plotted using the Tree-view software package [72]. *olSix3.2 *protein sequences were scanned for motifs using online software available at HGMP [73] and NCBI [74].

### Isolation of *olSix3.2 *cDNA

Total RNAs from medaka embryos at different stages were isolated by RNAzol B (Campro Scientific, Berlin, Germany) and treated with Dnase I (Invitrogen, Carlsbad, CA). RT-PCR reactions were performed using SUPERSCRIPT II (Invitrogen, Carlsbad, CA), as described previously [75]. PCR using *olSix3.2 *specific primers was performed using 2 μl of the reverse transcription reaction as a template with the High Fidelity PCR system (Roche, Basel, Switzerland). Oligonucleotide primers used to isolate *olSix3.2 *cDNA are listed in Additional data file 4.

### Plasmid constructions

A 4.5 kb region of *olSix3.2 *genomic sequence containing nine nucleotides (corresponding to the first three amino acids) of the coding region was cloned in frame with EGFP reporter gene into the pSKII-ISceI-EGFP vector [50], to create the cI construct. *Xenopus *and human sequences were amplified from corresponding genomic DNA and cloned in the pSKII-ISceI-EGFP vector with the same strategy. The medaka deleted constructs pSix3.2ΔXhoI (cII), pSix3.2ΔXhoI-NsiI (cVIII), and pSix3.2ΔXbaI-HindIII (cIX) were obtained by digesting the pSix3.2-4.5 kb construct using the indicated enzymes. All the other deleted constructs (pSix3.2Δel1, pSix3.2Δel2, pSix3.2Δel3, pSix3.2Δel4, pSix3.2Δel5, pSix3.2Δel6, and pSix3.2Δel7; cXXIII to cXXIX) were obtained by PCR amplification from pSix3.2-4.5 kb and then cloned into pSKII-ISceI-EGFP vector. The A, B, and C modules were deleted by restriction enzyme digestion (A, NarI/KpnI; B, BtsI/BglII; and C, BamHI/ClaI) and inserted (in sense and anti-sense orientations) into the polylinker of pSix3.2ΔXhoI (cIII to cV), pSix3.2Δel1 and pSKII-ISceI-Tk-EGFP (containing the tyrosine kinase minimal promoter) vectors to test their potential regulatory activity. All of the other modules were amplified and cloned (in sense and anti-sense orientations) into the polylinker of pSix3.2Δel1 (cVI to cVII, cX, and cXII to cXVI) and pSKII-ISceI-Tk-EGFP (cXI and cXVII to cXXII). The primer sequences used to generate these constructs are shown in Supplementary Table I. All constructs were verified by automated sequencing.

## Additional data files

The following additional data are available with the online version of this manuscript. Additional data file 1 is a Figure reporting the amino acid sequence alignment of *Six3 *genes from different vertebrate species. Additional data file 2 is a figure illustrating the phylogenetic tree of the *SIX *family. Additional data file 3 provides the precise nucleotide sequences of modules A to L described in the report. Additional data file 4 is a table listing the sequences of the primers used to amplify the DNA fragments, which were used to design the different constructs described in the report.

## Supplementary Material

Additional data file 1 Presented is a figure reporting the amino acid sequence alignment of *Six3 *genes from different vertebrate species Click here for file

Additional data file 2 Presented is a figure illustrating the phylogenetic tree of the *SIX *family Click here for file

Additional data file 3 Presented are the precise nucleotide sequences of modules A to L described in the report. Click here for file

Additional data file 4 Presented is a table listing the sequences of the primers used to amplify the DNA fragments, which were used to design the different constructs described in the report. Click here for file
