# Supplementary material for: Comprehensive characterization of the cis-regulatory code responsible for the spatio-temporal expression of olSix3.2 in the developing medaka forebrain
Source: Genome Biol. 2007 Jul 6;8(7):R137. doi: 10.1186/gb-2007-8-7-r137 (PMC2323233; doi:10.1186/gb-2007-8-7-r137)
Supplement: Additional data file 1 — Presented is a figure reporting the amino acid sequence alignment of Six3 genes from different vertebrate species [file gb-2007-8-7-r137-S1.ppt]

## Slide 1
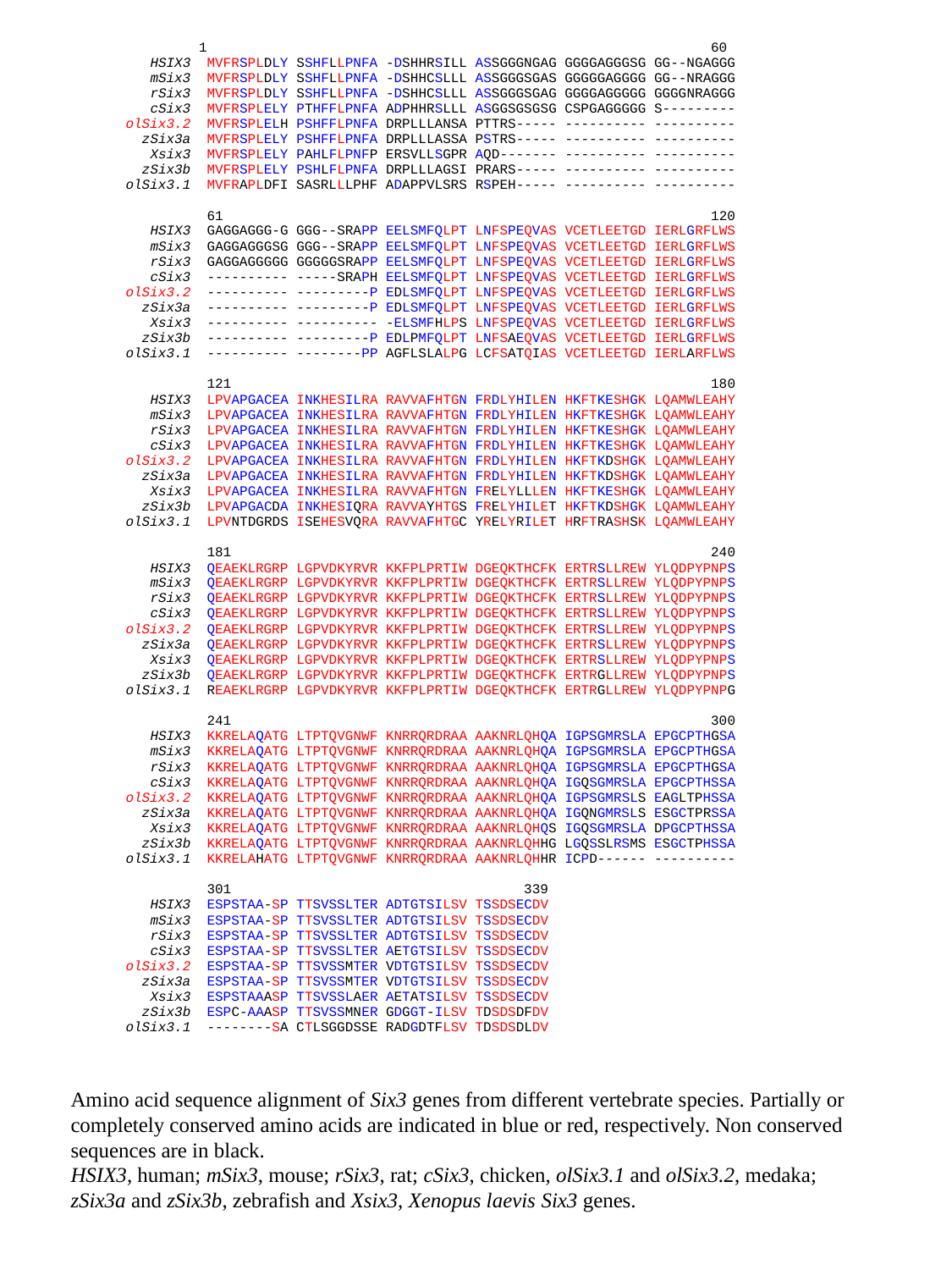

Amino acid sequence alignment of Six3 genes from different vertebrate species. Partially or completely conserved amino acids are indicated in blue or red, respectively. Non conserved sequences are in black.
HSIX3, human; mSix3, mouse; rSix3, rat; cSix3, chicken, olSix3.1 and olSix3.2, medaka; zSix3a and zSix3b, zebrafish and Xsix3, Xenopus laevis Six3 genes.
