# Supplementary material for: Comprehensive characterization of the cis-regulatory code responsible for the spatio-temporal expression of olSix3.2 in the developing medaka forebrain
Source: Genome Biol. 2007 Jul 6;8(7):R137. doi: 10.1186/gb-2007-8-7-r137 (PMC2323233; doi:10.1186/gb-2007-8-7-r137)
Supplement: Additional data file 2 — Presented is a figure illustrating the phylogenetic tree of the SIX family [file gb-2007-8-7-r137-S2.ppt]

## Slide 1
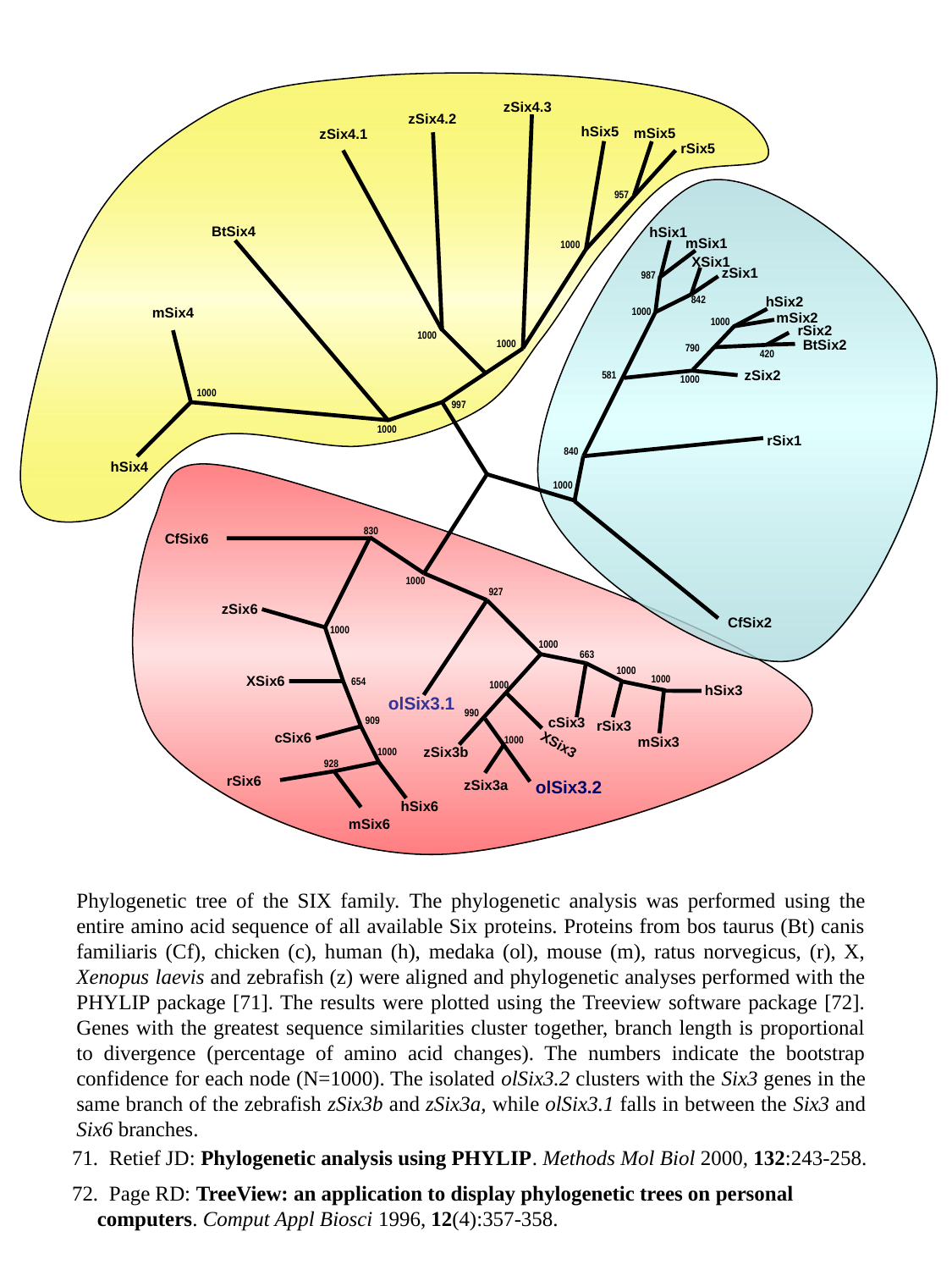

zSix4.3
zSix4.2
hSix5
mSix5
zSix4.1
rSix5
957
BtSix4
hSix1
mSix1
1000
XSix1
zSix1
987
hSix2
842
mSix4
1000
mSix2
1000
rSix2
1000
BtSix2
1000
790
420
zSix2
581
1000
1000
997
1000
rSix1
840
hSix4
1000
830
CfSix6
1000
927
zSix6
CfSix2
1000
1000
663
1000
XSix6
1000
654
1000
hSix3
olSix3.1
990
909
cSix3
rSix3
cSix6
mSix3
1000
XSix3
zSix3b
1000
928
rSix6
olSix3.2
zSix3a
hSix6
mSix6
Phylogenetic tree of the SIX family. The phylogenetic analysis was performed using the entire amino acid sequence of all available Six proteins. Proteins from bos taurus (Bt) canis familiaris (Cf), chicken (c), human (h), medaka (ol), mouse (m), ratus norvegicus, (r), X, Xenopus laevis and zebrafish (z) were aligned and phylogenetic analyses performed with the PHYLIP package [71]. The results were plotted using the Treeview software package [72]. Genes with the greatest sequence similarities cluster together, branch length is proportional to divergence (percentage of amino acid changes). The numbers indicate the bootstrap confidence for each node (N=1000). The isolated olSix3.2 clusters with the Six3 genes in the same branch of the zebrafish zSix3b and zSix3a, while olSix3.1 falls in between the Six3 and Six6 branches.
71. Retief JD: Phylogenetic analysis using PHYLIP. Methods Mol Biol 2000, 132:243-258.
72. Page RD: TreeView: an application to display phylogenetic trees on personal computers. Comput Appl Biosci 1996, 12(4):357-358.
