# Supplementary material for: Comprehensive characterization of the cis-regulatory code responsible for the spatio-temporal expression of olSix3.2 in the developing medaka forebrain
Source: Genome Biol. 2007 Jul 6;8(7):R137. doi: 10.1186/gb-2007-8-7-r137 (PMC2323233; doi:10.1186/gb-2007-8-7-r137)
Supplement: Additional data file 3 — Presented are the precise nucleotide sequences of modules A to L described in the report. [file gb-2007-8-7-r137-S3.doc]

BoxA

1 60

medaka CCTCATTAAA TGCCGCTAAC AACCCGTGTA AATACACTGA TTGGACAGCT CCGATACAAG

zSix3a CCTCATTAAA TGTCGCTAAC AAGCCGTGCA AATGCAGTGA TTGGACAGCT CCCGTATAAC

Fugu CCTCATTAAC TGTCGCTAAC AACCCGTGTA AATACACTGA TTGGACAGCT CCGATACAAG

Tetraodont CCTCATTAAC TGTCGCTAAC AACCCGTGTA AATACACTGA TTGGACAGCT CCGATACAAG

61 120

medaka CTCCAAGCCC TGCCAGGAAG TCCCCCTAAT GAGACCACAC AATCAAAAAC AATAAA----

zSix3a TCCCCAGACC TAGCAGGATG CCCACTTAAT GAGACCACAC AAAAA----- ----------

Fugu CTCCGAGCCC TGCCAGGAAG TGTCCCTAAT GAGACCACAC AATAAAAACA ATAATAGAAA

Tetraodont CTCCGAGCCC TGCCAGGAAG TGTCCCTAAT GAGACCACAC AATAAAAACA ATAATAGAAA

Box B

890 950

medaka GGATTGCACC GTCCCGATAG CATCACACAT CTGCTCTGCA GAGAAAAGAT CTAGTGGGTG

zSix3a GGAGAGCAAC GTCCCGGCAG AGGATGACAA CTTTAAAACA GATAAACTGG GAATATGCAT

Fugu AGTGCGCCCC GTCCCGACAG CATCACACAT CTACTCTTCA CAGACACACA GGCTGTTTGT

Tetraodont AGTGCGCCCC GTCCCGACAG CACCACACAT CTACTCTGCA CAAACACGCA GGCTGTTTTT

Box C

1441 1500

medaka ---------- ---------T CGGTTGGATC CAGGATG-GA GCAGCAG-CAC ATGAGTCTCC

Fugu GTCTGGTCCT GTGTATTGTT GAGACGAATT CAGGATG-GA GCAGCAGCCAC ATGAGTCGCT

Tetraodont GCCCGGTCCT GTGTATTGTT GAGATCAATT CAGGATG-GA GCAGCAGCCAC ACGAGTCGCT

1501 1560

medaka GCTCTGTTTG CCAGAGAGGG GTGGGCTGCC TTTT-CTGGC CAGGCTCTGC CAAACCGAGG

Fugu CCTGGGTTTG TCAGGG--GG GTGTGCTGCT TTTCACTAGA CAGCCTCAGT CAGACCGAGG

Tetraodont CCTG--TTTG TCGGGG--GT GTGTGCTGCT TTCCACTGGG CAATCTCAGT CAGACCGAGG

1561 1620

medaka TCATCGAGGG AGCAAAAACT GTAACAAAAC AAGAA----- ---------- ATGTTTTCTT

Fugu TCAACGAGGG AGCAAAATTA AATAAATAAT ACGGACATTC AAGCACAACA AGCCTTGATT

Tetraodont TCAACGAGGG AGCAAAATTA AATAAATAAT ACAAACATTC AAGCGCAATA AGCTTTGATT

1621 1680

medaka TTGCGCCGTC TTTCTCTTCC CTCTTACCTT TTCCAGTCGA CACCTTTT-- ---CTTTCTA

Fugu CCGCGCCTGC TT-------T CACTTTCTGA CAATAGTCAA CACCTTTTTA TTTCTTTCTG

Tetraodont ACGCGCCTCT TTCTCTTCCT GGCTTACCTT TAAAAGTCAA CACCTTTTTA TTTCTTTCTG

1681 1740

medaka TCCTCGTGCG CTCCCCTTCT CTCTCTCACT CTTATAAATG ACGTAACATC GGCTTTGTTT

Fugu TCATCAGGGG T--CCCTTCT CTCTCTCACT CC---ACATG ACGTAGCATC TGCTTTGTTT

Tetraodont TCATCGGGGG T--CCCTTCT CTCTCTCACT CC---ACATG ACGTAACGTC TGCTTTGTTT

1741 1800

medaka ATTTGCTCCC CTTCTAAGAA CAGACTG--- ---------- ---------- ----------

Fugu ATTTGCTGCC CTTATTGGAA CAGGAGTTAC TAATGTAATA ATTTAGAAAG CAACACATCA

Tetraodont ATTTGCTGCC CTTATTGGAA CAG-AGTTAC TAATGTAGTA ATTTACAAAG TAACACATCA

Box D

2041 2100

zSix3a ATTGCGAAGT CTTTGTCAGT ---------A AATAAACAAA GACGGAGCTA AACGTTCAGC

Fugu GTTGTCGAGC ATTTGGAGGT TTAAGGCGTA AGTAAACGGA GAAGAGCCTA AATGCGCGCT

Tetraodont GTTGTCGAGC TTTCGGAGGT TTAAGGCGTA AGTAAACGGA GAAGAGCCTA A-TGCGCGCT

medaka GTGTTGTTGC GTCTGGAGAT TTAGGCTGTG AGTAAACGAA GAAGACGCTA AATGCTCCGT

2101 2160

zSix3a GGTATTGAGG ACCTCGCTCC CTGCTGATTT TGGCATAGAA CAGAGCAGTG AAAGCTAGGG

Fugu GTGCATTCAG AGCTGCCGTC CCCCGCTGCT CGGAACATTT A--------G CAAGCCTACG

Tetraodont GTGCATTCAG AGCTGCCGTC CCCCGCTGCT CGGAACATTT A--------G CAAGCCTACG

medaka GCGCATTCAG AGCTCCCGTC CCCCGCTGCT AGGAACATTC A--------G CAAGCTCTCG

2161 2220

zSix3a GATAAACAGA CGATTAAACC AGGTGAATGA ACTTATTTGG GGA--TGCTA ATTACTTGCT

Fugu GATAAACACA CAATCAGGGC AGGTGAATAA ACTTTTAGGG GGGGGTGTTA ATTACATGCT

Tetraodont GATAAACACA CAATTAGGGC AGGTGAATAA ACTTGTTTGG GGG--TGTTA ATTACATGCT

medaka GATAAACACA CAATTACGGC AGGTGAATAA ACTTGTTTGG GGAG-TGTTA ATTACGTGCT

2221 2280

zSix3a TAAAGAACAC AAAGGTTATG TTAAACAAAG TGATTTAACA A--------- ----------

Fugu TAACGTAAAC AAAAGGCGTG TTTAAGAATG TGAATTACCA GTGGCTTTTC C--TTCAATC

Tetraodont TAACTTAAAC AAAAGGCATG TAAAAGAATG TGAATTACCA GTGGCTTTTC T--CTCAATC

medaka TGACGTAAAC AAAAGCCATG TAAAAGAATG TGAATTAGCT GTGGCTTTCC TGCCTCAATC

BoxE

2581 2640

zSix3a ---------T TATTTCCTGT CCAAAAAGCT TTCCAAAGGG ACCATTTCAA GTGCCACTTG

Fugu TACCAGCCTT TACTTCCAGT CCACAGAGCT TTCCACCGGG ACCAATTCAA GTGCCACTTG

Tetraodont TACCAGCCTT TACTTCCAGT CCACAGAGCT TTCCACCGGG ACCAATTCAA GTGCCACTTG

medaka TACCAGCCTT TACTTCCAGT CCAATGAGCT TTCCAATGGG ACCAAATCAA GTGCCACTTA

2641 2700

zSix3a GAATTACTTC ACTTAAGTTT CCTGTGACAG AAGAACAAAC AGATTTGATC CTGGAAA---

Fugu GAATTAGTCC TCTTGGTGTT CGTATAGCAG TGCAGCGGAC AGGTTTGATC CCACACAGCC

Tetraodont GAATTAGTCC TCTTGGTGTT CGTATAGCAG TGCAGCGGAC AGGTTTGATC CCACACAGCC

medaka GAATTAGTCC TCCTGAATTT TGTGCGGCAG CACAGTGGAC AGGTTTGATC CC--------

Box G

2941 3000

zSix3a ---------- ---ATTTTTA AATAATCAGA TGAACTCCTA GGTACCTATT TGAATTCAAG

Fugu CCCAGTCCCG GAGAATGTCA AAAATCTGAG AGAGCGAGAA GGAAAAGTTT TGAATTCCTG

Tetraodont CCCGGTCCCG GGGAATGTCA AAAATCTGAG GGAG--AGAA GGAAAAGTTT TGAATTCCTG

medaka ---------- ---------- ---------- ---------- ---AAAGTTT TGAATTCCTG

3001 3060

zSix3a ACC------- TGGAAGAGAA ATTTAGGACT TTAACCAATC GTGGAAAATG CAGAAAATAA

Fugu TATGATTTGG TAGAAAGGCA GCTTAGGATC ATTATGTCAG CTTTATTGAG GACGGATTAA

Tetraodont AATGATTTGG TAGAAAGGCA GCTTAGGATC ATTATGTCAG CTTTATTGAG GACGGATTAA

medaka AATGAATTTG TAGAAAGGC- GCTTAGGATC ATTATGTCAG CTTTATGGAG GACGGATTAA

3061 3120

zSix3a TTTTAGCATT AAGATGAAGT AAGTCTCTCG CCTCTTATTG TTCTCACAAT ATGTTCTTGA

Fugu GCCGAGTCTA GATGCTGCGC CGTGCACGGC CCTTTCATAA TGTGAGCCCT GTTCTGTTG-

Tetraodont GCCGAGCCTA GATGCTGCGC CGTGCACGGC CCTTTCAGAA TGCGAGCCCT GTTCTGTTG-

medaka GCGGAGTCTA GATGCTACGC CTTGTACACG GTTCTCTCAC AATGTGATCA CTGTTCGTTT

3121 3180

zSix3a TCGAGTGAAC GCGCACAAAT TTAACAGCAA CTAAGCACTA TTTAAATATT ATTCAGCACA

Fugu --GAGCGGAC TCACACTATT TTAACACCCT GTAAGCCCTG TCTAAATATG AAGCCGCCTT

Tetraodont --GAGCGGAC TCACACTATT TTAACACTCT GTAAGCCCTG TCTAAATATG AAGCCGTCTT

medaka TATAGTGAAC CCACACTATT TTAACACCTT GCAAACCCTG TCTAAACATG AAACAGTTTC

3181 3240

zSix3a AACTATTTGA TACAAACCAG GAAGCCCATT CCAGCACG-- --CTCCTGCA AGCCGAACCC

Fugu AGCCAT---- ----AAACAT GAAAATCACA CCGCCACGGC TACTGGTGTA AGCCGAACCG

Tetraodont AGCTAT---- ----AAACAT GAAAATAACA CCGCCACGGC CACCGGTATA AGCCGGACCG

medaka AGCCAT---- ----AAACAT GAAAATCACA CCGCTTTGGC TG

Box H

3250 3310

Fugu TAAGCCGAAC CGTGTGACTG GACGA----G GACATTGATC ACGTCCTTAC --CCTAAATC

Tetraodont TAAGCCGGAC CGTGTGACTG GATGT----G GACATTGATC ACGTCCTTAC --TCTAAATC

medaka TAAGCCAAAT CCTGTGACTG GATGCATGGG GACATTGATG TTGTCCTTAT AGCCTCAGAT

3311 3370

Fugu AAACATAAAA ACATGGCTTT TATGTCCCCA AGAAGCTTTG TGTTGCCATT TAATTGCAAT

Tetraodont AAACATAAAA AC--GGCTTT TATGTCCCCA CGGAGCTTTG TGCTGCCATT TGATTGCAAT

medaka CAAATGCAAG AAACTGCTTT TATGTGCCTA GTATCTTTTA TGTTGCCATT CGATTGTAAT

Box I

3021 3080

zSix3a ---------- --TACATATC TTCTTAA-CT TTAACGAGCC TCGTTAAGAT CACAATAATA

Fugu GCATCTCCAG TCTACATATC TTCTTTAGCT TTAACGAGAC TCGTTAAGAT CGCAATAATA

Tetraodont GCATCTCCAG TCTACATATC TCCTTTTGCT TTAACGAGAC TCGTTAAGAT CGCAATAATA

medaka GCATCTCCAG TCTACATATC TTCTTTAGCT TTAACGAGCC TCGTTAAGAT CGCAATAATA

3081 3140

zSix3a TTCCACCCAC TAATTGCTCA TTCCATTCAA CAAATAGGCG AGAGTCTGCT TCGACTTCAC

Fugu TTCCACCCTC TAATTGCTCA TTCCATTCAG CAGATAGGCG AGCATTGCCT TGCGCCTGAT

Tetraodont TTCCACCCTC TAATTGCTCA TTCCATTCAG CAGATAGGCG AGCGCTGGCT CGCGCCCGAT

medaka TTCCACCCTC TAATTGCTCA TTCCATTCAG CAGATAGGCG AGCATTGGCT TGTGCCTGAT

3141 4200

zSix3a ACGAGTCAA- --GGGAGGGA GGGAGCTGTT GAGATTGGAG TTCCCGATAA CCCCCCGTGC

Fugu GCGGTGCGGT GCGGTGGGAG GGTTGCTGTG GAGATTGGAG ACTCTGATAA CCCCCCGTGC

Tetraodont GCGCTGCGCT GCGGTGGGAG GGTTGCTGTG GAGATGGGAG ACTCTGATAA CCCCCCGTGC

medaka GCGC-GCGGT GCGGTGGGAG GGTTGCTGTG GAGATCCTAG ACTCTGATAA CCCCCCGTGC

Box L

4201 4260

zSix3a GCAGCCGAAG TGGTGAAAGC CTC----TAC GTACTGGCTA ATGATTGGCA CGCTTGACAG

Fugu GTTGCACAAG TGGTGAAAGC CTCGCGCTAC GTACTGGCTA ATGATTGGCA CGCTTGACAG

Tetraodont GTTGCACAAG TGGTGAAAGC CTCGCGCTAC GTACTGGCTA ATGATTGGCA CGCTTGACAG

medaka GCTGCACAAG TGGTGAAAGC CTCGCGCTAC GTACTGGCTA ATGATTGGCA CGCTTGACAG

4261 4320

zSix3a TGATTGGCAG GGCTGCCATG ACAACCCTAC AACGACACCG AGAAGACCAA TAGAAAAGCG

Fugu TGATTGGCAG GGCTGCCATG ACAACGCTAC AACGACACCA AGAAGACCAA TAGAAAAGGG

Tetraodont TGATTGGCAG TGCTGCCATG ACAACGCTAC AACGACACCA AGAAGACCAA TAGAAAAGGG

medaka TGATTGGCAG GGCTGCCATG ACAACGCTAC AACGACACCA AGAAGACCAA TAGAAAAGGG

4321 4380

zSix3a AAACAAAATA TTTCAATGCT ACACTCAC-- GGTGGATTTA GGGGGAGATA TTATGAGGCT

Fugu AAACAAAATG TTTCAATGCT ACACTCAACA GCGGATTTAG GGGGGAGATA TTATGAGGCT

Tetraodont AAACAAAATG TTTCAATGCT ACACTCAACA GCGGATTTAG GGGGGAGATA TTATGAGGCT

medaka AAACAAAATG TTTCAATGCT ACACTCAACG GCGGATTTAG GGGGGAGATA TTATGAGGCT

4381 4440

Six3a GGTGTCATTA GGCGATAGCT ATTGAATCAT TCAATCTGAA TTCGTCG--- ----------

Fugu GGTGTCATTA GGCGATAGCC ATTGAATCAT TCAATCTTTT TTACTAGCCG TGTTTTTTCG

Tetraodont GGTGTCATTA GGCGATAGCC ATTGAATCAT TCAATCTTTT TTACTAGCCG TGTTTTTTCG

medaka GGTGTCATTA GGCGATAGCC ATTGAATCAT TGAATCTTTT TTACTTACCG TATTTTTC
