# Supplementary material for: Comprehensive characterization of the cis-regulatory code responsible for the spatio-temporal expression of olSix3.2 in the developing medaka forebrain
Source: Genome Biol. 2007 Jul 6;8(7):R137. doi: 10.1186/gb-2007-8-7-r137 (PMC2323233; doi:10.1186/gb-2007-8-7-r137)
Supplement: Additional data file 4 — Presented is a table listing the sequences of the primers used to amplify the DNA fragments, which were used to design the different constructs described in the report. [file gb-2007-8-7-r137-S4.doc]

**Table 1**

olSix3/4kbF CCTCATTAAATGTCGCTAAC

olSix3/4kbF1 TGCAGTGATTGGACAGCTCC

olSix3/5UTR/R cgcctaatgacaccagcctc

olSix3/5UTR/R1 ccctgccaatcactgtcaag

olSIX3R GTAAAGCTCGCGATAGCATC

olSIX3R1 CCATATGGTTCTGGGTAAGG

Six3/4kbNotI/F AAATgcggccgcCAACCCGTGTAAATACAC Six3/4kbNotI/R CTCTGCGGCCGCTCTGAAAACCATGGAAATG

hSix3tard/SalI/F CTACTGTCGACGAATCCATTCAACATATAC

hSix3/ATG/NotI/R GTCTAGGGCGGCCGCGAATACCATGGACTG

Six3.2DelX-XhoI/F GTGCTCGAGTTCCAGTGTTGGAAC

Six3.2DelXSalIdaXhoI/R CTTAGCAGGTCGACTCCGAATATG

Six3.2DelSalIdaXhoI/F CATATTCGGAGTCGACCTGCTAAG

Six3.2DelSalIdaXhoI/RN3 TGAAAGTGTCGACATCTGGTGGTG

Six3.2DelSalIdaXhoI/F2 ACTCACTGTCTGTCGACAGTGAGG

Six3.2DelSalIdaXhoI/RN2 CCCTCACTGTCGACAGACAGTGAG

Six3.2SalIEN1F ATGTCGACCACACCGCTTTGGCTG

Six3.2SalIEN1/Rnested ATGCATCCAGTCGACGGATTTGGC

Six3.2SalIEN1/R TCCCAATTTAAACAGGTCGACTGG

Six3.2 Del1/SalI/F GTGCAGGTCGACTTGCTTCCATTC

Six3.2 Del2/SalI/F CTCTACTAAGCATCTCCAGTCGAC

Six3.2 Del3/SalI/F AATAATAGTCGACCCTCTAATTGC

Six3.2 Del4/SalI/F GGTTGCTGTCGACATCCTAGACTC

Six3.2 Del5/SalI/F GATTGGCACGGTCGACAGTGATTG

Six3.2DelY/SalI/R CCAATCACTGTCAAGCGTGCCAATC

Six3.2DelY/SalI/R2 CGCCGTCGACTGTAGCATTGAAAC

Six3.2DelY/SalI/R3 ATGGCTGTCGACTAATGACACCAG

EGFP/R GGCAGTTTGCCGGTGGTGCATATG

Six3/CNS484/F aaaagttttgaattcctcaatg

Six3/CNS484/F1 attatttcagctttattgagg

Six3/CNS486/R ttCTGTAACTATGGAAGTGTG

XenSix3/F AGTAGTTTGAATTCCTGAATG

XenSix3/R TCTAGAGGAGACCTGAACACCATG

Six3.2 Del2/SalI/R GTCGACTGGAGATGCTTAGTAGAG

Six3.2 Del3/SalI/R GCAATTAGAGGGTCGACTATTATT

Six3.2 Del4/SalI/R GAGTCTAGGATGTCGACAGCAACC

Six3.2Kpn/F AACTATGCAATAGTTCAATATC

List of the primers used to amplify some of the different medaka genomic regions used to engineer the different construct used in the course of this study
